# Early Emergence of Adaptive Mechanisms Sustaining Ig Production: Application to Antibody Therapy

**DOI:** 10.3389/fimmu.2021.671998

**Published:** 2021-04-29

**Authors:** Maud Lemarié, Fabrice Chatonnet, Gersende Caron, Thierry Fest

**Affiliations:** ^1^ Université de Rennes 1, INSERM, Établissement Français du Sang de Bretagne, UMR_S1236, Rennes, France; ^2^ Laboratoire d’Hématologie, Pôle de Biologie, Centre Hospitalier Universitaire, Rennes, France

**Keywords:** UPR, ER stress, B cell differentiation, mAbs, RNA-seq

## Abstract

Antibody therapy, where artificially-produced immunoglobulins (Ig) are used to treat pathological conditions such as auto-immune diseases and cancers, is a very innovative and competitive field. Although substantial efforts have been made in recent years to obtain specific and efficient antibodies, there is still room for improvement especially when considering a precise tissular targeting or increasing antigen affinity. A better understanding of the cellular and molecular steps of terminal B cell differentiation, in which an antigen-activated B cell becomes an antibody secreting cell, may improve antibody therapy. In this review, we use our recently published data about human B cell differentiation, to show that the mechanisms necessary to adapt a metamorphosing B cell to its new secretory function appear quite early in the differentiation process i.e., at the pre-plasmablast stage. After characterizing the molecular pathways appearing at this stage, we will focus on recent findings about two main processes involved in antibody production: unfolded protein response (UPR) and endoplasmic reticulum (ER) stress. We’ll show that many genes coding for factors involved in UPR and ER stress are induced at the pre-plasmablast stage, sustaining our hypothesis. Finally, we propose to use this recently acquired knowledge to improve productivity of industrialized therapeutic antibodies.

## On the road to Become an Ingenious Secreted-Antibody Factory: Differentiation Steps From B to Plasma Cell

Plasma cells (PCs) secrete huge amount of immunoglobulin molecules (Igs) subsequently to antigen entry into the body. Before becoming high-affinity antibody secreting cells (ASCs), B cells undergo several steps of differentiation. First, inside the bone marrow, precursor B cells edit a B-cell receptor (BCR) (or surface-attached IgM, an-antigen specific Ig of the first line of defence with poor affinity towards the antigen). At this point, they produce Ig but only intended to be transmembrane receptors. Naive B cells (NBCs) are in a resting state in peripheral blood or secondary lymphoid organs until their activation by a foreign antigen. Once activated by circulating antigens, NBCs reach a secondary lymphoid organ and move towards the B: T interface where they receive help from specialized CD4+ T cells called follicular helper T cells (Tfh) *via* efficient B: T synapses (1–3). B cells need interaction with several co-activators, including CD40L and the delivery of cytokines including IL-21 and IL-4, to undergo their differentiation into fully mature effectors. The terminal steps of the differentiation occur in a microanatomical specialized area of secondary lymphoid organs called germinal centers (GCs) which are created by B cells themselves in response to *BCL6* expression. In this context, IL-21 represents the main upstream cytokine responsible for BCL6 maximal expression and GCs maintenance (4). GCs are organized into two separated territories - called light zone and dark zone – between which the B cell continuously moves until reaching a high affinity for targeted antigens (1–3). At first, B cells proliferate in the dark zone where cells undergo AID-driven somatic hypermutation (SHM) of variable regions of their Ig gene loci. The second step takes place into the light zone where B cell clones carrying a modified variable region of Ig are tested for its antigen affinity by follicular dendritic cells with the help of Tfh cells. Clonal B cells go through this step with 4 different outcomes based on the strength of BCR signal (antigen affinity) and the amount of Tfh help received: (i) a low-affinity and no help leads to apoptosis of the clone; (ii) mid-affinity and low Tfh help leads to the formation of a long-lived memory cell, (iii) higher affinity and T cell help leads to another round of SHM in the dark zone and (iv) highest levels of both signal leads to the differentiation into a long-lived plasma cell (PC) (2, 3, 5). This B cell maturation is completed by the Ig class-switch recombination (CSR), allowing cells to produce and secrete IgM, IgG, IgE or IgA, each class offering specific functions to adapt the antibody response to the context.

**Figure 1:**
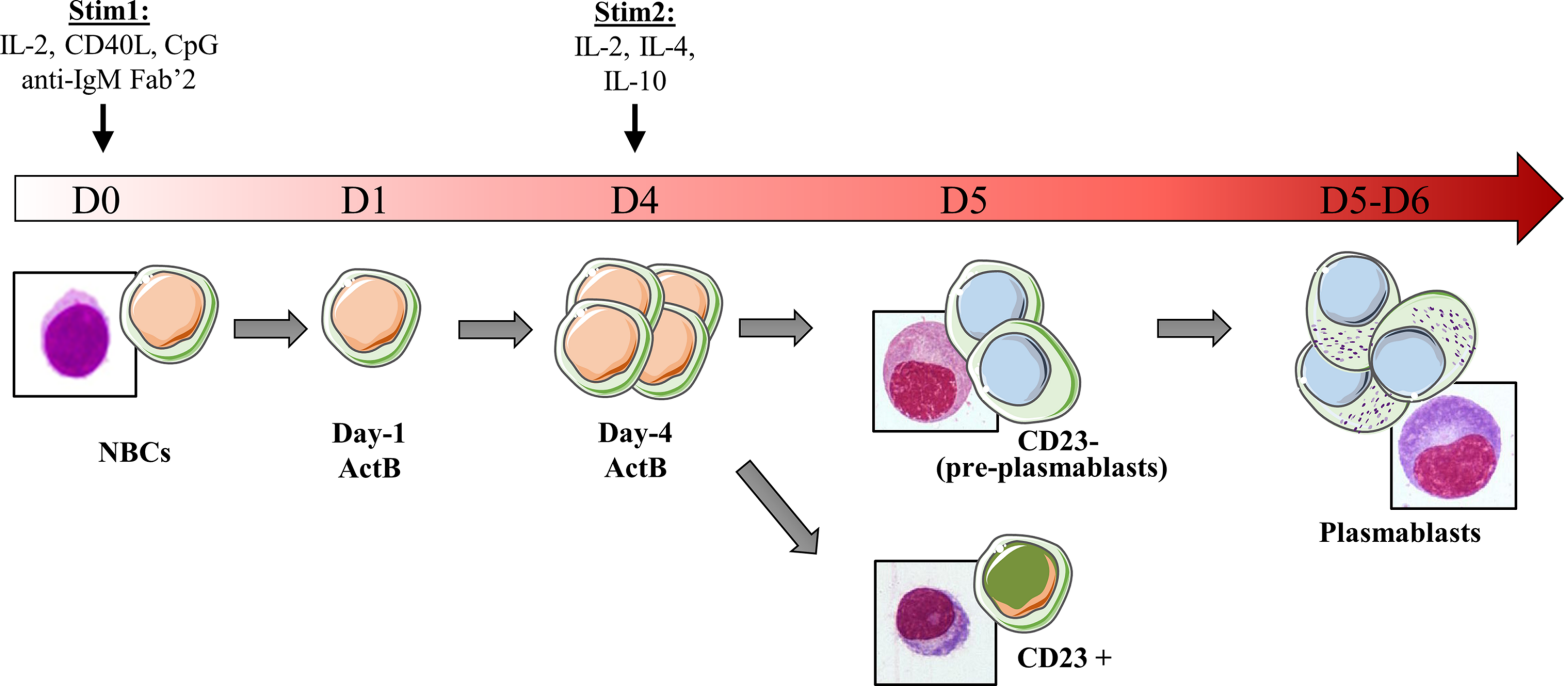
Schematic representation of the *in vitro* model of B cells differentiation used in our laboratory. Peripheral NBCs from blood donors are stained with a cell-tracer then stimulated with IL-2, CD40L, CpG and anti-IgM Fab’2. Day-1 activated cells are referred as day-1 ActB. After 4 days, activated B cells that have proliferated (Day-4 ActB) are selected according cell-tracer dilution and stimulated with IL-2, IL-4 and IL-10 to induce their differentiation into plasmablasts. The day after (D5), three populations are detected: (i) CD23+ cells that are stuck in an activated state and unable to differentiate, (ii) CD23- population, containing precursors of plasmablasts (pre-PB) which give rise to (iii) differentiated plasmablasts (PB). The increase in the cytoplasm/nucleus ratio, characteristic of the development of the Ig production machinery in PB, is early detected in the pre-PB stage. RNA-seq data are available for NBC, Day-1 ActB, Day-4 ActB, CD23+, CD23- and PB subsets.

In our lab, we developed and standardized an *in vitro* model system of human NBCs differentiation into plasmablasts (PBs) (**Figure 1**). Starting from blood donor buffy coat we purify NBCs and then culture them with IL-2, CD40L, CpG and anti-IgM Fab’2 in order to activate cells *via* a transcriptional burst (6). As soon as day-1, B cells are fully activated and referred hereafter as day-1 ActB. Beyond day-4 (day-4 ActB), culture conditions are modified and cells maintained only with IL-2, IL-4 and IL-10 stimulation for 2 or 3 additional days in order to complete the PB differentiation. We showed recently that committed B cells that differentiate into PBs present an extinction of both IL-4/STAT6 signaling and CBLB ubiquitin ligase expression, concomitant to IRF4 induction (7). As a surrogate marker of this commitment, membrane surface expression of CD23 disappears due to IL-4/STAT6 extinction. We showed that day-5 CD23^-^ post-ActB cells contain precursors of plasmablasts (pre-PBs) which present the capacity to enter the cell cycle, while the CD23^+^ counterparts are unable to differentiate and stay in an activated state (7, 8). After nearly 7-8 days of culture, some PBs start to express the PC-specific CD138 marker which points out the generation of early PCs. PB and PC differentiated *in vitro* secrete high amounts of Igs; they display morphological and transcriptomic features of their *in vivo* counterpart and represent an useful tool to explore normal human PB and PC biology (8–10).

While becoming a PC and therefore fully efficient for Ig secretion, cells experience massive organelles modifications including membrane amplification and trafficking. The endoplasmic reticulum structure reaches its maximum of protein production during this stage (11) and cells are subject to an intensive and continuous stress that needs to be controlled to escape from cell death.

## Early Appearance of Adaptive Mechanisms to High Throughput Ig Secretion

### General Mechanisms of ER Stress and Unfolded Protein Response (UPR)

In every tissue, cells continuously produce proteins in their cytoplasm to meet their needs and to respond to microenvironment signals. Igs are part of the transmembrane and secreted proteins. After being transcribed, mRNA is pushed to the surface of the endoplasmic reticulum (ER) to be processed by the ER-attached ribosomes and translated into the ER lumen. As the translation occurs in the ER, proteins enter the lumen in their misfolded form. From here, Igs produced in naive B cells start their journey through multiple organelles in order to be well-processed and then bound to the membrane. After antigen activation, IgGs that are produced in PBs/PCs are aimed to be secreted outside of the cell.

**Figure 2:**
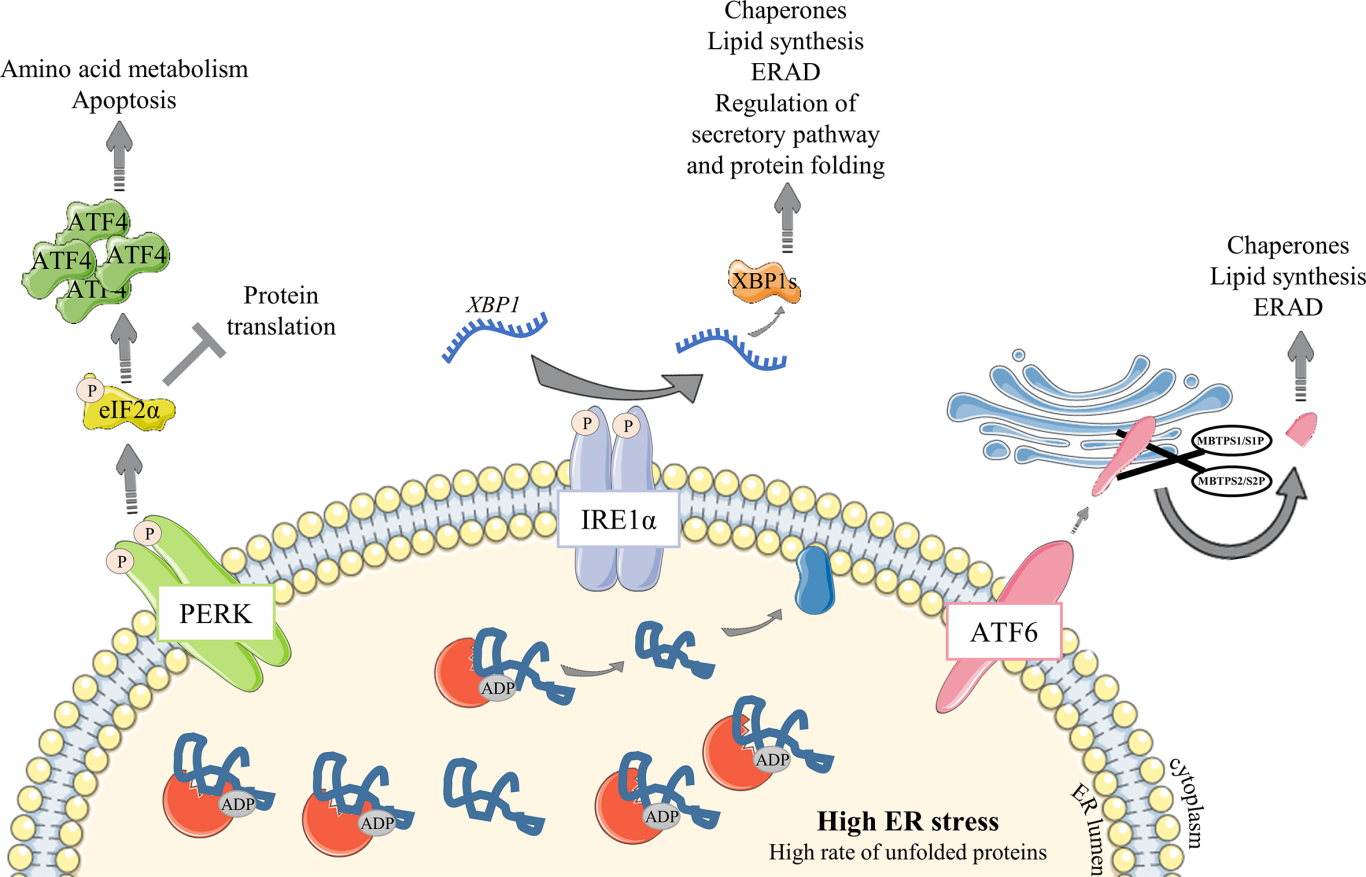
Overview of the UPR signaling pathway under ER stress conditions. Unfolded Protein Response is engaged to respond to increasing amount of proteins in the ER. (1) The ER-resident chaperone BiP binds to unfolded and misfolded proteins and consequently releases the sequestrated IRE1α, PERK and ATF6 ER sensors, leading to their activation following (i) dimerization and auto-phosphorylation of IRE1α and PERK elements or (ii) *MBTPS1*/S1P and *MBTPS2*/S2P – mediated cleavage of ATF6α in the Golgi apparatus. Activated ATF6α then translocates into the nucleus and upregulates chaperones expression and factors involved in lipid synthesis and ERAD. Activated PERK phosphorylates eIF2α which represses global protein translation except for ATF4 whose translation is enhanced. ATF4 then upregulates amino acid metabolism and apoptosis. IRE1α, *via* XBP-1 transcription factor, leads to a broader range of responses with upregulation of many factors including chaperones and those important for lipid synthesis and ERAD. IRE1α regulates as well the factors important for protein folding and secretory functions. Particularly, *XBP1* mRNA requires IRE1α to be spliced and efficient as a transcription factor.

After antigen encounter and B cell differentiation into PC, a metabolic switch occurs and a sharp increase in nutrient uptake is necessary to meet the growing need (reviewed in (12)). Ig processing is overwhelmed into the ER lumen, leading to an unfolded and misfolded protein (hereafter designated un/misfolded protein) rate increase and an ER high-stress state. The unfolded protein response (UPR) is then engaged in these cells to meet the increasing needs for protein processing, folding and secretion, and to protect them from apoptosis. Three well-characterized UPR axes have been described as involved in ER stress response: (i) inositol-requiring trans-membrane kinase/endonuclease 1α (IRE1α)/X-box binding protein (XBP) - 1, (ii) protein kinase RNA-like endoplasmic reticulum kinase (PERK) and (iii) activating transcription factor 6 (ATF6) pathways (13, 14). In resting conditions, the ATPase Ig-binding protein (BiP) - a resident-ER chaperone - binds to each of them. Stress signals then lead to a BiP UPR first-line of response to ensure activation of ER transmembrane IRE1α, PERK and ATF6 elements. Indeed, BiP dissociates from IRE1α and PERK proteins to release them and then binds to un/misfolded proteins (15). Importantly, BiP association/dissociation cycle with IRE1α and PERK is governed by an ADP-ATP cycle. ATP-bound BiP preferentially binds to ER transmembrane elements (16). When ER stress is increasing, DnaJ-like co-factors (ERdj) bind to un/misfolded proteins and accompany their transfer to the ATP-bound BiP, leading subsequently to ATP hydrolysis (17, 18). As a consequence, BiP dissociates from ER elements and then associates with un/misfolded proteins under its ADP-binding state, stably sequestering clients to prevent aggregation in the ER lumen and accompanying for folding (18, 19). BiP-clients complex dissociation, induced by Nucleotide Exchange Factors (NEF) which remove ADP from BiP, is then required to properly complete folding process and secretion. BiP consequently returns to its initial position with ER elements, meaning that ER stress is under control (20, 21). ATF6 is also released from BiP during ER stress (22) but needs an additional step to be fully activated. Hence, the released ATF6 is then cleaved in the Golgi apparatus by Site-1 and Site-2 proteases (encodes by *MBTPS1* and *MBTPS2*, respectively) before being translocated back to the nucleus to induce the expression of UPR genes such as *XBP1* (23–26) (**Figure 2**).

### Early Appearance and Temporal Regulation of ER Stress and UPR Factors

**Figure 3:**
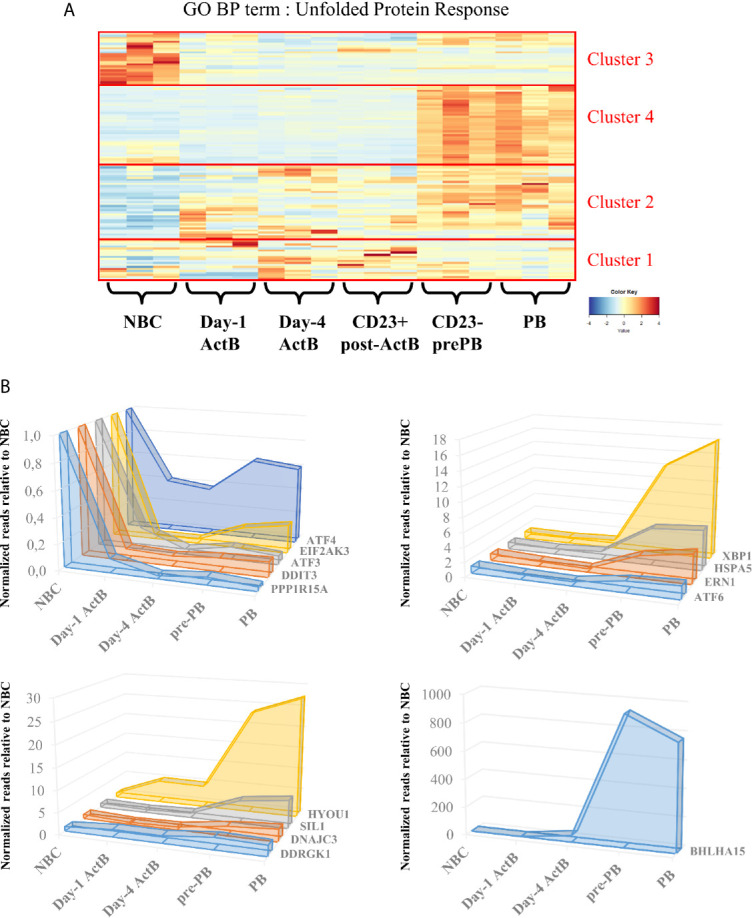
Unfolded protein response is temporally regulated during the transition from naive B cells to plasmablasts. **(A)** Genes belonging to the Gene Ontology – Biological Processes term “Unfolded Protein Response” were selected and their expression in our RNA-seq data was submitted to an unsupervised hierarchical clustering analysis. The resulting heatmap is shown here, delineating four different expression clusters: (1) stable expression, (2) genes upregulated from Day-1, (3) genes upregulated in NBC and (4) genes upregulated in Pre-PB and PB. **(B)** Means of normalized reads obtained from 3 different experiments at Day-1 ActB, Day-4 ActB, pre-PB and PB stages were compared to the NBC counterpart, whose mean is reduced to 1. Left upper panel shows genes related to the PERK pathway. Right upper panel shows genes related to the IRE1α and ATF6 pathways and lower panels show potential genes which are either implicated (i) in the PERK regulation or (ii) in endoplasmic reticulum modifications related to stress. All of the selected genes are addressed in this review.

ER stress responses seem to be engaged well before PCs massively produce antibodies. A preparation to morphological and genomic modifications that NBCs undergo to become ASCs is probably needed. During B cell differentiation, BLIMP-1 (encoded by *PRDM1*), the major regulator of the antibody-secretory function of PCs, represses the B cell identity transcriptional program including genes encoding for BCL6 and PAX5 (27), and enhances *XBP1* expression (28). Activation of this latter factor is one of the most important events and is maintained essentially by BLIMP-1. Interestingly, BLIMP-1 positively controls the activity of mammalian target of rapamycin (mTORC1) (29), which is described as a key metabolic factor since it controls proteins, nucleotides and lipids synthesis (30). mTORC1 has also been well characterized in B cell development (31, 32) but its role in ER stress has emerged only in the last decade with a few studies showing its importance for a sustainable Ig production. mTORC1 inhibition in mature murine PCs leads to a decrease in serum IgM and IgG levels and a failure to induce BiP protein expression (33, 34). More recently, mTORC1 was defined as a precocious factor in the UPR response to Ig production and secretion. In fact, two separated studies unveiled crucial aspects of mTORC1 function in murine PCs. Double knock-out of XBP-1 and TSC1, a mTOR inhibitor, leads to an increase of Ig production and the differentiation into PCs was maintained even in absence of XBP-1 (33). These results suggest the existence of an alternative UPR response independent of the IRE1α/XBP-1 and ATF6 pathways. Additional studies indicate that UPR-, protein production- and secretion-affiliated gene expressions - such as *Hspa5*, *Pdia6*, *Ero1l*, encoding respectively for BiP, for a protein disulfide isomerase and for an ER-resident oxidase, increased in activated B cells dependently of mTORC1. Interestingly, this upregulation is observed well before the activation of BLIMP1-dependent PC program and *Xbp1* gene expression in mice (35). RNA-seq data obtained throughout our *in vitro* differentiation model of human B cells (7) support these results and show an UPR response well before cells are secreting antibodies. Hence, bio-informatic analyses of the RNA-seq dataset segregated genes linked to UPR by GO annotations into 4 clusters according to gene expression modifications during the differentiation steps: 1) stable from NBCs to PB stage; 2) upregulated as early as the day-1 ActB stage, 3) downregulated right after B cell activation, and 4) upregulated in pre-PB and maintained in PBs (**Figure 3A**). Indeed, the comparison between NBCs, day-1 ActB, day-4 ActB, post-ActB, pre-PB cells, and PB cells shows that UPR-affiliated genes seem to be only partially influenced by the *XBP-1* and *PRDM1* expression levels. Since cluster 4 includes XBP-1, IRE1α and ATF6α factors, we hypothesized that UPR-associated genes specifically upregulated in pre-PB are IRE1α/XBP-1- or ATF6-dependent which is not the case for genes from cluster 2. The main functions associated with each cluster were then evaluated by the DAVID pathway enrichment software in order to find differences between ER stress response governed or not by IRE1α/XBP-1 or ATF6. Thus, genes from cluster 4 whose expression is specifically upregulated in Pre-PB and PB stages are mainly associated with IRE1α - and ATF6- mediated UPR, protein folding and transport, and apoptosis inhibition. For genes involved in cluster 2 whose expression appears as soon as day-1 of the culture, they are mainly associated with ER to Golgi vesicle-mediated transport, ER stress suppression and IRE1α -mediated UPR (a few genes compared to cluster 4). Overall, both UPR and ER stress responses are activated early in B cells engaged in PC differentiation and in at least two separate phases with different molecular requirements.

### A Specific Inhibition of the PERK Pathway Throughout B Cell Differentiation

In contrast to insulin-secreting pancreas cells (36–38) and collagen-secreting chondrocytes (39), Ig secretion is described as using a PERK-independent ER stress response (40–42). In our *in vitro* model, *EIF2AK3* (which encodes PERK itself) but also well-characterized *DDIT3* (also known as CHOP) and *ATF4*, both pro-apoptotic PERK target genes, are all included in cluster 3. Interestingly, when the gene expressions in ActB and PB populations are compared to NBCs we noticed that in addition to *EIF2AK3* and *ATF4*, other specific genes of the PERK pathway such as *ATF3* and *PPP1R15A* are part of the cluster 3 (**Figure 3A** and **Table S1**). Their expressions are 2 to 10 times higher in NBCs compared to the more mature stages (**Figure 3B**). In contrast, the IRE1α/ATF6 target gene, *HSPA5* (from cluster 4) maintains high levels of expression from NBC to ActB stages and even increases its expression (by more than 3 times) at pre-PB stage (**Figures 3B**). Altogether, these data show that PERK-pathway gets inactivated as soon as NBCs are stimulated and remains inhibited in PC.

**Figure 4:**
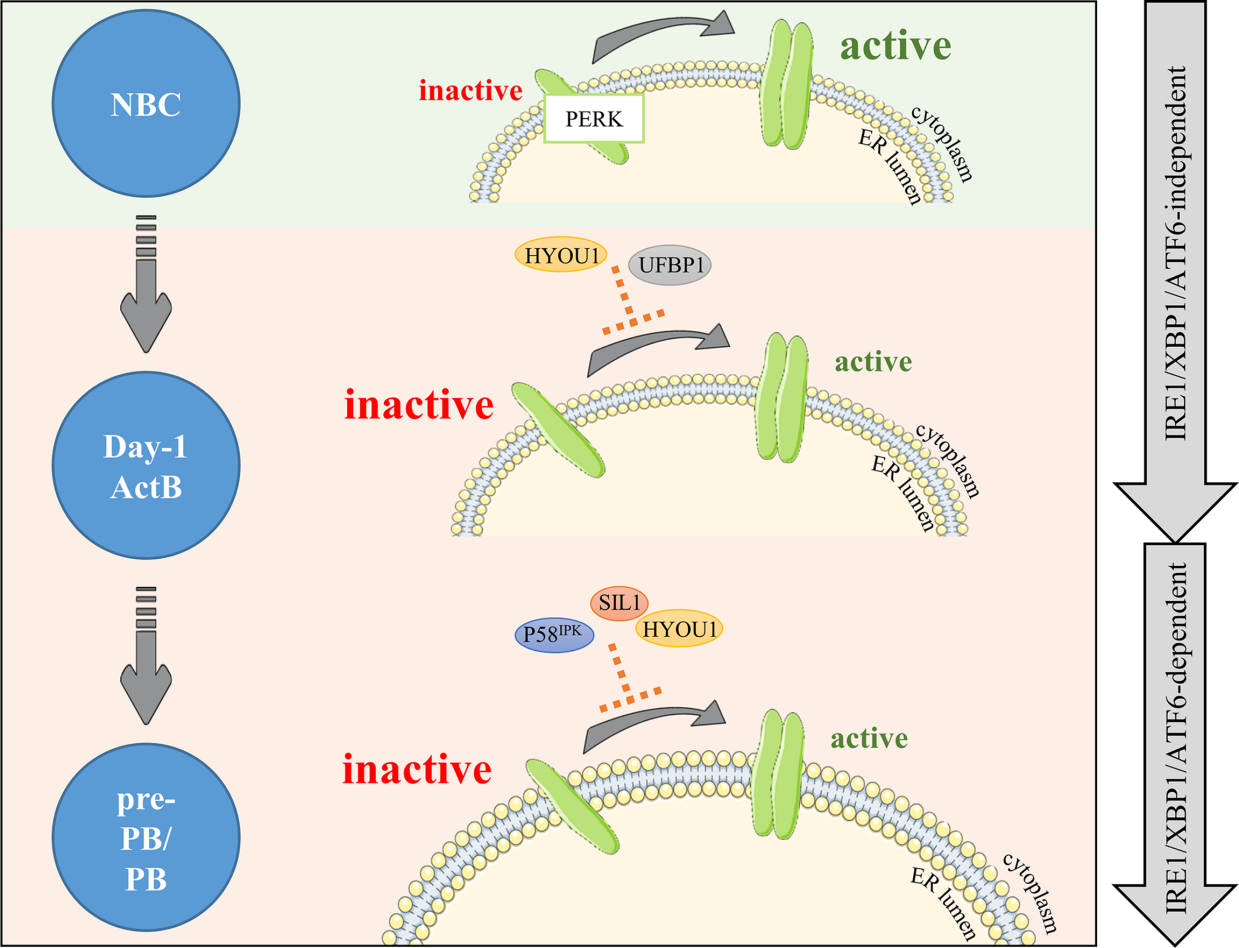
PERK is negatively regulated right after B cell activation and maintained under negative control during differentiation. PERK signaling is upregulated in NBCs (light green background). In activated B cells, PERK needs to be dampened by factors limiting PERK activity (light orange background). Our RNA-seq analysis combined with literature emphases on two new potential negative factors: HYOU1 and UFBP1. Then, differentiation (pre-PB/PB) involves important changes including increased production of Ig, which critically increases ER stress and risks of apoptosis; hence, PERK signaling repression needs to be reinforced. Factors potentially involved in this control are part of the ADP/ATP BiP regulation cycle: the DnaJ protein P58^IPK^ and the nucleotide exchange factors SIL1 and HYOU1. The first wave of negative control is IRE1α/XBP-1/ATF6-independent and the second wave is IRE1α/XBP-1/ATF6-dependent (right gray arrows).

Characterization of the factors involved in the suppression of the PERK pathway during PC differentiation has long been elusive. However, two recent studies demonstrated the role of UFBP1 (Ufm1 binding protein 1; issued from the gene *DDRGK1* presents in cluster 2) in this process. Indeed, UFBP1-mediated ufmylation of IRE1α protein protects from IRE1α degradation, leading to its stabilization and the suppression of the PERK pathway (43). In addition, the UFBP1-mediated suppression of PERK leads to the active promotion of PC differentiation and therefore ER expansion (44). Since the expression of *DDRGK1* occurs in cluster 2 in our *in vitro* model, we speculate that UFBP1-mediated PERK inhibition is primarily IRE1α/XBP-1 and ATF6 independent (**Figures 3**, **4** and **Table S1**). However, *DDRGK1* expression increases slightly in pre-PB and PB populations suggesting that a delayed IRE1α/XBP-1 and ATF6 effect may exist and participate in *DDRGK1* expression at latter stages of differentiation.

The PERK pathway is also actively suppressed by the P58^IPK^ protein in many cell types. Mechanistically, P58^IPK^ binds directly to the kinase domain of PERK, which impedes its activity and further activation of its targets (45). P58^IPK^ is encoded by *DNAJC3*, a member of the DnaJ family acting downstream of the ATF6 and IRE1α/XBP-1 pathways (28, 45–50). Although no clear evidence exist so far for its role in B cells, some studies showed that p58^IPK^ protein expression increased rapidly after stimulation of murine B cells with LPS (41, 42) or CpG (35), while the expression of PERK downstream targets decreased. In our model of human B cell differentiation, the expression of *DNAJC3* increases strongly in cluster 4 in pre-PB and PB populations compared to ActB cells. This is in agreement with Gaudette et al. study in mice (35), which suggested that P58^IPK^ could partially contribute to the inhibition of the PERK pathway in ASCs, as soon as in the pre-PB stage (**Figures 3**, **4** and **Table S1**).

Similarly, SIL1, a nucleotide exchange factor, could represent another potential inhibitor of PERK since the *SIL1* knockdown in HeLa cells showed an activation of the PERK pathway (51). In our model, *SIL1* expression was strongly induced in pre-PB and PB (around 5-fold increase) compared to the earlier differentiation stages. Although *SIL1* does not belong to the GO term UPR in Biological Processes (BP), we found it in the RE Cellular Component (CC) annotation. A similar temporal clustering of our RNA-seq data performed on genes of this GO term showed that *SIL1* belongs to a cluster of genes over-expressed specifically in pre-PBs and PBs (GO CC: RE; cluster 3) (**Figures 3B**, **S1** and **Table S2**). Interestingly, SIL1 has been described as a co-chaperone of BiP required for the release of BiP-clients in the ER lumen (20, 21) after sequestration with BiP. However, the knockdown mouse model of Ichhaporia et al. demonstrated that SIL1 was unnecessary for the release of BiP from un/misfolded Ig and for subsequent production of Ig (52). Recently, ChIP-sequencing data done in human PBs (GSE142493 (53);) revealed an enrichment of XBP-1 in *SIL1* promoter, suggesting a potential role of SIL1 in cell differentiation. This result, together with the HeLa data described above and our model of B cell differentiation strongly suggests that SIL may play a role in the inhibition of the PERK pathway in human ASCs (**Figure 4**). In addition, HYOU1 (also known as ORP150) which is another nucleotide exchange factor for BiP, can substitute for SIL1 (54–56) and has been described in the literature as a cyto-protective factor against ER stress (57, 58). In our system, *HYOU1* (which, unlike *SIL1*, is included in the GO BP UPR term) belongs to the cluster 4 and its expression is (i) higher than for *SIL1* and (ii) strongly upregulated in pre-PB and PB stages (**Figure 3** and **Table S1**). Then, given that HYOU1 can substitute for SIL1 in its functions, HYOU1 could represent an alternative factor of suppression of PERK, active in the last stages of B cell differentiation (**Figure 4**).

Taken together, these data provide support to a better understanding of PERK suppression in ASCs. Consistent with previous studies (35, 42), we found that the PERK pathway is inhibited early in the process of B cell differentiation by the specific repression of some of its genes. Two phases of repression seem to exist: (i) a strong first wave of repression, IRE1α/XBP-1 and ATF6 independent which appears right after NBC activation, and (ii) a second wave, dependent on IRE1α/XBP-1 and ATF6 occurring when Ig production is at its highest level (**Figure 3B**). ASCs are constantly in a state of prolonged and elevated ER stress and therefore at a constant risk of apoptosis. Even though the implication of apoptosis mediated by the PERK and IRE1α/XBP-1 pathways in the resolution of acute ER stress is controversial (31, 59–70), prolonged PERK signaling promotes apoptosis in contrast to a sustained IRE1α response which improves cell survival (71). Therefore, in order to achieve successful antibody secretion in ASCs, it appears that PERK signaling needs to be restricted. In addition, previous studies suggested that while PERK could be partially activated in stimulated B cells, the subsequent signal appears not to be sufficient to induce target genes (41, 42).

Interestingly, the expression pattern of *HYOU1* is bimodal with the first and second peak of expression corresponding, respectively, to the first and second wave of PERK inhibition (**Figures 3B** and **4**). Since XBP-1 and ATF6 have been reported as critical inductors of *Hyou1* expression in murine B cells and fibroblasts, respectively, we hypothesise that both factors may contribute to HYOU1-mediated PERK inhibition in the late stage of B cell differentiation (35, 49). However, to date, no mechanism of action has been highlighted regarding either HYOU1 or SIL1 suppression of PERK pathway and further investigations are required to clarify this point. Altogether, HYOU1, in addition to provide ER stress protection (72–74), may exhibit critical repressor control of PERK in ASCs together with UFBP1 and P58^IPK^.

### Focus on MIST1, a Factor Involved in IRE1α/XBP-1/ATF6 Response

In the literature, XBP-1 appears to be a central factor governing the UPR response in PC differentiation and Ig secretion. To be actively efficient under ER-stress conditions, XBP-1 mRNA needs to be spliced by IRE1α which is previously released from BiP and then activated after auto-phosphorylation (75). Among potential XBP-1 target genes identified in murine PCs, MIST1 (encoded by *Bhlha15*) is a factor which has already been documented as important in secretory functions of some cells (76–80). *Bhlha15* promoter is bound by XBP-1 in murine PCs (76) and its expression is under XBP-1 dependence in several cell types including PCs (76, 81, 82). The study published by Capoccia et al. (83) ascribed a potential role of MIST1 in ER stress occurring in PCs. By transcriptome comparison between day-3 LPS-treated PCs obtained from *Bhlha15* ^-/-^ and WT mice, they found among the 218 differentially expressed genes specific functional annotations for endoplasmic reticulum and molecules transport. PCs associated with the small intestine have shown dilated and unorganized rough ER supporting the fact that MIST1 plays a critical role in ER stress despite its lack of involvement in PC differentiation (83).

In mice, the expression of *Bhlha15* like *Xbp1* is strongly induced in PCs compared to NBCs, MBCs and GC B cells (83, 84) (GSE 4142). In addition, *Bhlha15* ^-/-^ mice present a significant decrease of specific antibodies secretion in the serum 7 days after immunization (84). Overall, MIST1 and its respective functions have only been studied in the late stages of PC differentiation. Our RNA-seq data give a more precise picture on the regulation of *BHLHA15* expression during normal human B cell differentiation and notably by showing an expression as soon as day-4 of the culture (**Figure 3B**), prompting for further studies about its function in ER-stress and UPR management during B cell differentiation.

## Using B Cell Differentiation Mechanisms to Improve Immunoglobulin Production

Production of recombinant protein has been well studied for the past few decades and research continues to constantly improve this engineered production. Among recombinant proteins, monoclonal antibodies (mAbs) production reports largely to the pharmaceutical industry. The first industrialized mAb was approved in 1997 and used for non-hodgkin’s lymphoma patients. Rituxan^®^, better known as Rituximab, was employed to recognize CD20 on B cells as an antigen. Almost 80 approved therapeutic mAbs were developed since, such as Humira^®^ (Adalimumab/anti-TNFα), Xolair^®^ (Omalizumab/anti-IgE), Opdivo^®^ (Nivolumab/blocks PD-L1 binding to PD-1 and PD-2) or more recently Sarclisa^®^ (Isatuximab/anti-CD38) and Imfinzi^®^ (Durvalumab/blocks PD-L1 binding to PD-1 and CD80). Each of them involved an intensive work mainly focused on how to efficiently increase mAb productivity (mAb titer and quality), a major concern in industrial settings.

Chinese Hamster Ovary (CHO) cells are the main producing cell line for engineered monoclonal antibody production. CHO cells are easy to manipulate and enable post-translational modifications important for Igs functions, such as glycosylation. Production is organized as (i) CHO cell line generation, consisting in the development of the vector for recombinant protein expression and its transfection into CHO cells, (ii) selection/purification of clones which integrated the vector, (iii) large scale bioproduction and (iv) final formulation to appropriately administrate mAbs into the patient. Continuous productivity improvement could be achieved by modifying cell culture conditions (85–87) and editing genes involved in protein translation, folding and secretion.

Massive production of recombinant proteins in CHO cells leads to an important ER stress requiring control to attain a production with high stability and quality. Uncontrolled ER stress generates less secreted proteins (mainly due to apoptosis of overstressed clones), but can be regulated by inducing autophagy (88). It also leads to a final product of poor quality (aggregates and subvisible particles coming from protein folding and assembly dysfunctions) (89), which, for the latter, represents issues for further clinical use (90). Hence, recent works proposed to control factors involved in the UPR pathway since some of them were described as able to monitor and control ER stress during Ig production. For instance, Talbot et al. (91) proposed to monitor ER stress in order to control aggregates concentration into the final product. To this end, they used two different culture conditions based on the presence or absence of specific nutrients for production of two different Ig subtypes. The deprivation condition induced a higher and earlier ER stress. The increase in the relative gene expression of several UPR-specific genes was assessed throughout culture and revealed a specific signature assigned to each Ig subtype and culture condition. Using this signature to monitor cell cultures, the authors obtained a significant decrease in aggregates and subvisible particles for IgG1 mAbs, unlike what was observed for IgG2 mAbs. In the case of IgG1 production, *HSPA5* was induced later than for IgG2 while *DERL3*, an ER-associated degradation (ERAD)-specific gene which helps for un/misfolded protein degradation (92–94) is induced earlier. This protected cells from overwhelmed UPR and allowed an extended time of culture. With the support of other studies, detailing ER biomarkers profiling during of CHO cell lines culture (95–97), they finally proposed to include a UPR genes signature to the quality parameters of mAbs production, providing culture conditions labelled as “ER stress under control”. As an example, *HERPUD1*, another ERAD-specific gene, is described as an early indicator of ER stress response (96, 97) and then could be used to predict production efficiency and stability. Interestingly, this gene is included in the cluster 2 of our study, indicating that it is an early UPR marker during the B cell differentiation process. Hence, our RNA-seq dataset supports conclusions of published data (95–97) and provides new potential factors to detect i) when ER stress is precociously managed, with genes such as *VCP* or *MBTPS2* (both in cluster 2), and ii) UPR intensity during culture of high recombinant protein expressing cell lines with *SYVN1*, *SSR1, DERL1, DERL2, MBTPS1* or *WFS1* (all included in cluster 4).

The UPR activation is cell line-dependent but appears to be also clone-dependent. In fact, producing high-proliferative clones with high quality specificities is possible but relays on their proper ability to activate UPR and to escape from apoptosis. Therefore, a clonal selection process is necessary to select for clones with the best performance in terms of mAb titers, cell mass and viability. To this aim, UPR can be monitored with different UPR-inducible systems in CHO cells by (i) using promoters containing all three UPR responsive elements (UPRE, ERSE, and ACGT) (98), (ii) using native promoters of ER-stress induced factors such as BiP (99) or (iii) using a fluorescent reporter which produce specific fluorescence based on *XBP-1* splicing by activated IRE1α (96). Although those systems allow for selection of clones with the best performance, Poulain et al. (100) showed recently that during selection, the lower the expression of the protein of interest is in a pool of cells, the higher will be the frequency of clones with high productivity in this pool. To show this, they transduced a CHO cell line containing an inducible expression system called cumate-gene switch with an inducible plasmid containing the gene of interest. This system allowed to study the impact of high- versus low-expressing ones on final productivity. Therefore, reducing expression of recombinant proteins in CHO cells combined with selection of clones with higher capacities to deploy UPR, increases final recombinant protein titers and stability.

Decreasing ER stress during recombinant protein production has been one of the main concerns in mAb research since a few years. Therefore, controlling ER stress could represent a complementary aspect to the monitoring one. Work mainly focused on UPR-specific genes activation has been successfully done with factors such as IRE1α (101), BiP (102, 103) or XBP-1 (104). PDIs (protein disulfite isomerases) which are upregulated during Ig production (such as *PDIA5* and *PDIA6* in cluster 4; **Figure 3A**) have also been shown to increase the protein secretion rate in CHO cells when overexpressed (105, 106).

**Figure 5:**
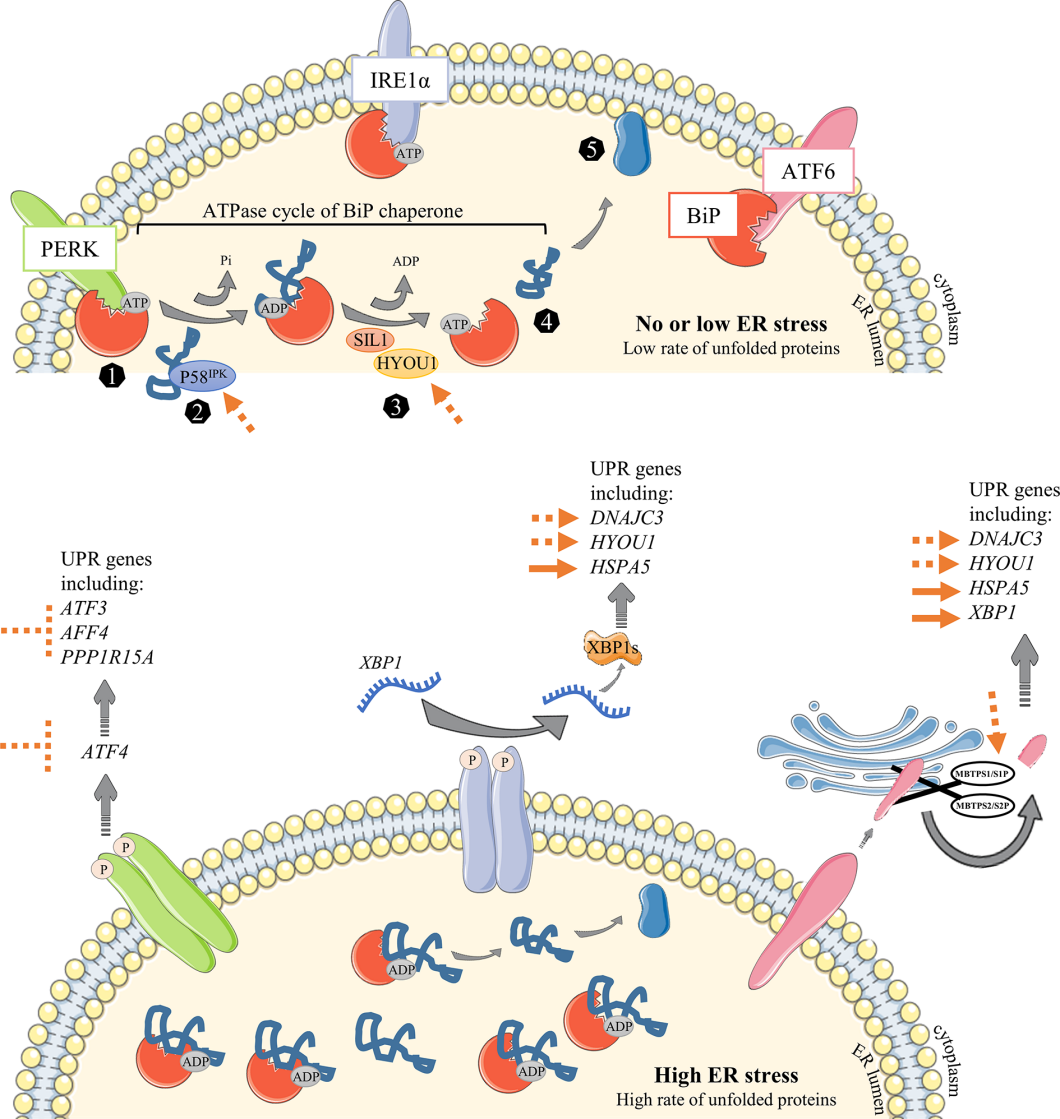
Overview of known or suggested strategies to improve Ig production in CHO cells. In the first steps of NBC activation, a low rate of proteins is processed into the ER leading to no or negligible stress conditions (upper panel). When cells undergo the following steps of differentiation that will result in the generation of highly-producing secreted Ig PBs/PCs, mass of their ER is getting bigger and associated with an important stress. Unfolded Protein Response is then engaged to cope with the increasing amount of proteins in the ER. (1) The ER-resident chaperone BiP binds to unfolded and misfolded proteins and consequently releases the sequestrated IRE1α, PERK and ATF6 ER sensors, leading to their activation following (i) dimerization and auto-phosphorylation of IRE1α and PERK elements or (ii) *MBTPS1*/S1P and *MBTPS2*/S2P – mediated cleavage of ATF6α in the Golgi apparatus. (2) DnaJ family members such as P58^IPK^ (encoded by *DNAJC3*) bind directly to unfolded and misfolded proteins to guide their transfer onto an ATP-bound BiP. Given the ATPase activity of BiP, an inorganic phosphate (Pi) is instantly released from BiP, leading to a conformational modification which stabilizes protein into the ADP-bound BiP (3). Thereafter, nucleotide-exchange factors (NEFs) such as SIL1 and HYOU1 facilitate the release of ADP from BiP and the rebinding of ATP. 4-5) As a consequence, client is released and then processed to be secreted outside of the cell. The main strategies already proposed to improve Ig production in CHO cells are represented as full orange arrows. The strategies discussed in this review are represented as repressive or permissive dotted orange arrows.

As previously described in this review, BiP, XBP-1, IRE1α and PDI are part of the cluster 4 in the dataset. Importantly, many other factors whose expression follows cluster 4 pattern are UPR sensors. Among them, P58^IPK^, MIST1 (both described in chapter 2), DERL1, WFS1, or even MBTPS1/S1P (**Figure 5**) could represent new targets to improve ER stress response during the production of mAbs. As previously shown, some pathways relative to ER stress responses are engaged very early after B cell activation, as shown by the cluster 2 in **Figure 3A**. Therefore, it would be advantageous to study genes from cluster 2 such as *DDRGK1* (UFBP1 protein, described in chapter 2), *PIGBOS1*, *HERPUD1*, *DCTN1*, *MBTPS2/S2P* or *ERO1A* in order to increase the number of potential novel engineering targets. HYOU1, whose expression pattern has been discussed in this review, could be another interesting target (**Figure 5**), since its overexpression was shown to improve ER stress responses in liver cells from obese diabetic mice (74) or neurons under hypoxia (72, 73).

Nonetheless, inhibiting UPR suppressors such as ATF6β (107) could also represent an alternative way to increase mAbs productivity. Our analysis identifies *DNAJB9* from cluster 4 as a potential new target since it acts by inhibiting activation of IRE1α (108). Moreover, playing with PERK signaling could offer new strategies to improve productivity. Thus, the control of PERK downstream targets such as *ATF3*, *DDIT3* or directly *ATF4* or *EIF2AK3* in CHO cells can protect cells from apoptosis (**Figure 5**). In accordance with our hypothesis, Roy et al. (96) observed a PERK inhibition feedback coupled with IRE1α activation in cells with high levels of IgG secretion after several days of culture and argued that a mechanism may exists in PCs to decrease apoptosis risk over production time.

## Conclusion

The model used in our laboratory facilitates the study of B cell development from NBCs to the plasmablast stage and allows to visualize at molecular level any modifications occurring either early or late during the maturation process (6–8, 109–111). Indeed, this paper brings new elements concerning the regulation of PERK and some factors that could have an impact on the productivity of mAbs. PERK regulation is poorly studied in B cells and our present study merges RNA-sequencing with already published data regarding ER stress response in other cell types to unveil new mechanisms that need to be further studied in B cells. Additionally, despite the fact that research in mAbs engineering has been intensive over the past decades, here we have opened up new perspectives for the optimization of mAbs production.

## Author Contributions

ML performed the literature search, wrote and revised the manuscript, designed the figures and analysed the clusters with the DAVID bio-informatic tool. FC analysed the RNA-sequencing dataset, generated heatmaps and revised the manuscript. GC and TF revised the manuscript. All authors contributed to the article and approved the submitted version.

## Funding 

This study was funded by Agence Nationale de la Recherche (ANR) number: ANR-19-CE15-0020-01.

## Conflict of Interest

The authors declare that the research was conducted in the absence of any commercial or financial relationships that could be construed as a potential conflict of interest.
