# Supplementary figures and images for: Early Emergence of Adaptive Mechanisms Sustaining Ig Production: Application to Antibody Therapy
Source: Front Immunol. 2021 Apr 29;12:671998. doi: 10.3389/fimmu.2021.671998 (PMC8117215; doi:10.3389/fimmu.2021.671998)

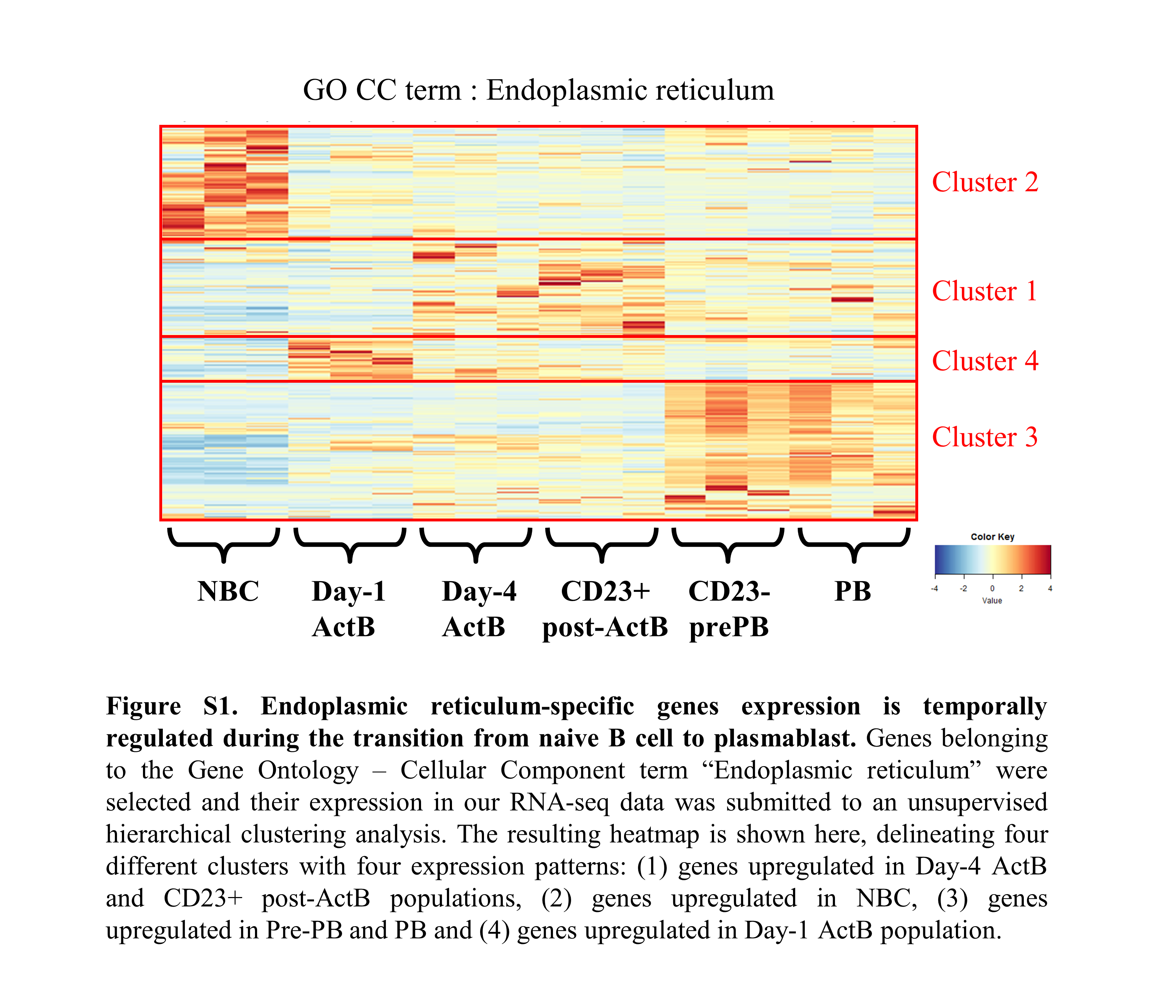

Supplement: Supplementary file 1 [file Image_1.tif]
